# Delayed Administration of a Bio-Engineered Zinc-Finger VEGF-A Gene Therapy Is Neuroprotective and Attenuates Allodynia Following Traumatic Spinal Cord Injury

**DOI:** 10.1371/journal.pone.0096137

**Published:** 2014-05-20

**Authors:** Sarah A. Figley, Yang Liu, Spyridon K. Karadimas, Kajana Satkunendrarajah, Peter Fettes, S. Kaye Spratt, Gary Lee, Dale Ando, Richard Surosky, Martin Giedlin, Michael G. Fehlings

**Affiliations:** 1 Department of Genetics and Development, Toronto Western Research Institute, and Spinal Program, Krembil Neuroscience Centre, University Health Network, Toronto, Ontario, Canada; 2 Institute of Medical Sciences, University of Toronto, Toronto, Ontario, Canada; 3 Department of Therapeutic Development, Sangamo BioSciences, Pt. Richmond, California, United States of America; 4 Department of Surgery, University of Toronto, Toronto, Ontario, Canada; Rutgers-Robert wood Johnson Medical School, United States of America

## Abstract

Following spinal cord injury (SCI) there are drastic changes that occur in the spinal microvasculature, including ischemia, hemorrhage, endothelial cell death and blood-spinal cord barrier disruption. Vascular endothelial growth factor-A (VEGF-A) is a pleiotropic factor recognized for its pro-angiogenic properties; however, VEGF has recently been shown to provide neuroprotection. We hypothesized that delivery of AdV-ZFP-VEGF – an adenovirally delivered bio-engineered zinc-finger transcription factor that promotes endogenous VEGF-A expression – would result in angiogenesis, neuroprotection and functional recovery following SCI. This novel VEGF gene therapy induces the endogenous production of multiple VEGF-A isoforms; a critical factor for proper vascular development and repair. Briefly, female Wistar rats – under cyclosporin immunosuppression – received a 35 g clip-compression injury and were administered AdV-ZFP-VEGF or AdV-eGFP at 24 hours post-SCI. qRT-PCR and Western Blot analysis of VEGF-A mRNA and protein, showed significant increases in VEGF-A expression in AdV-ZFP-VEGF treated animals (p<0.001 and p<0.05, respectively). Analysis of NF200, TUNEL, and RECA-1 indicated that AdV-ZFP-VEGF increased axonal preservation (p<0.05), reduced cell death (p<0.01), and increased blood vessels (p<0.01), respectively. Moreover, AdV-ZFP-VEGF resulted in a 10% increase in blood vessel proliferation (p<0.001). Catwalk™ analysis showed AdV-ZFP-VEGF treatment dramatically improves hindlimb weight support (p<0.05) and increases hindlimb swing speed (p<0.02) when compared to control animals. Finally, AdV-ZFP-VEGF administration provided a significant reduction in allodynia (p<0.01). Overall, the results of this study indicate that AdV-ZFP-VEGF administration can be delivered in a clinically relevant time-window following SCI (24 hours) and provide significant molecular and functional benefits.

## Introduction

In North America, it is estimated that approximately 1.5 million individuals are currently living with SCI, with over 12,000 traumatic SCI cases occurring each year [1]. Spinal cord injury is divided into two events, to separate the physical and the cellular pathologies. The primary injury, is associated with the initial mechanical trauma that the cord undergoes, whereas the secondary injury refers to the physiological cascade that propagates from 1 minute to 6 months following the initial injury [2]. Although the primary injury is responsible for triggering all of the downstream events, it is widely accepted that the processes that take place in the “secondary injury” phase are predominantly responsible for a significant portion of the damage and degeneration that is associated with SCI, including inflammation, ischemia, lipid peroxidation, production of free radicals, disruption of ion channels, necrosis and programmed cell death [3]–[5]. Moreover, radical alterations to the spinal microvascular architecture and function occur following SCI and contribute to the secondary injury. Reduction in blood flow, hemorrhage, systemic hypotension, loss of microcirculation, disruption of the blood-spinal cord barrier (BSCB) and loss of structural organization, ultimately enhance the cellular damage post-injury [2], [6]. Despite the fact that these secondary events are responsible for the majority of the damage associated with SCI, many of these pathways alternatively provide an opportunity to target with therapeutic interventions.

Recently, research has given much attention to therapies designed at repairing or minimizing vascular damage following injury. Angiogenic factors, such as vascular endothelial growth factor (VEGF)-A, are known to promote the proliferation of endothelial cells and initiate angiogenesis [7]. Emerging evidence suggests that VEGF-A (which will be referred to as VEGF) also has neurotrophic, neuroprotective, and neuroproliferative effects [8]. VEGF is a homodimeric glycoprotein that is expressed as multiple splice variants encoded by a single gene; however, VEGF signals as a homo- or heterodimer via VEGF receptors (VEGFRs) [9]. The predominant isoforms in the central nervous system are VEGF_121_, VEGF_165_ and VEGF_189_. Studies have demonstrated that VEGF and its receptors are upregulated during and after hypoxic/ischemic injury to the brain and spinal cord, which suggests that VEGF likely plays a neuroprotective (or beneficial) role in these pathophysiological processes.

Perhaps the most devastating outcomes of spinal cord injury are paralysis and neuropathic pain. Paralysis is caused by damaged axons and neurons in motor pathways at or above the level of injury. Many models of SCI have been used to model the physical deficits post-injury, and thoracic injuries are among the best-characterized for the targets loss of hindlimb function. Motor impairment following SCI results from damage to and/or loss of both upper and lower motor neurons. Injury to first and second order spinothalamic neurons, or first order neurons from the medial lemniscus pathway, interrupts sensory information processing *at* and *below* the level of injury and prevents normal signal transmission to the brain. Miscommunication in sensory pathways can result in severe complications for patients suffering from SCI. Development of neuropathic pain occurs in many patients, and although the exact mechanism is unknown, it is hypothesized that it is caused by misguided axonal sprouting or abnormal sodium channel excitability in sensory neurons [10].

Previously described approaches using VEGF have relied on the introduction of a single splice isoform of VEGF-A (VEGF_165_), which may not result in optimal neuroprotective or angiogenic effects. In this study, we utilize novel ZFP-VEGF technology – a viral vector encoding a zinc-finger transcription factor protein (ZFP), which activates endogenous VEGF-A expression to produce multiple splice isoforms of VEGF – which has previously demonstrated induced expression of VEGF-A protein, increase vascular counts and significant functional recovery following SCI [11]. Although we have already shown beneficial effects of AdV-ZFP-VEGF when administered immediately following SCI as a proof-of-concept, the current study aims to investigate a clinically-relevant administration of AdV-ZFP-VEGF by ***delaying*** administration by 24 hours post-SCI.

## Materials and Methods

All animal experiments were conducted with approval from the Animal Care Committee, University Health Network (Toronto, Canada).

### Viral Vector Constructs

The VEGF-A-activating ZFP and controls were provided in viral vectors by Sangamo BioSciences (Pt. Richmond, CA) and have been previously described [12], [13]. The VEGF-A-activating ZFP (32E-p65) – referred to as AdV-ZFP-VEGF – is a 378 amino acid multi-domain protein that is composed of three functional regions: (1) the nuclear localization signal (NLS) of the large T-antigen of SV40, (2) a designed 3-finger zinc-fingered protein (32E) that binds to a 9 base-pair target DNA sequence (GGGGGTGAC) present in the human VEGF-A promoter region and (3) the transactivation domain from the p65 subunit of human NFκB, which is identical to VZ+434, subcloned into pVAX1 (Invitrogen, San Diego, CA) with expression driven by the human cytomegalovirus (CMV) promoter. Adenoviral (Ad5-32Ep65 or Ad5- eGFP) vectors, referred to as AdV-ZFP-VEGF and AdV-eGFP, respectively, were packaged by transfecting T-REx-293 cells (Invitrogen, San Diego, CA). T-REx-293 cells in ten-stack cell factories were inoculated with Ad vectors at a multiplicity of infection (MOI) of 50 to 100 particles per cell. When adenoviral mediated cytopathy effect (CPE) was observed, cells were harvested and lysed by three cycles of freezing and thawing. Crude lysates were clarified by centrifugation, and 293 cells were seeded at 4×10^7^ PFU and grown 3 days prior to transfection. The calcium phosphate method was used for transfection. Infectious titers of the Ad vectors were quantified using the Adeno-X Rapid Titer kit (Clontech, Mountain View, CA).

### SCI and Intraspinal Microinjection

Animals were subject to a compressive spinal cord injury using a modified aneurysm clip, which has been extensively characterized by our laboratory and previously described [14]. Briefly, adult female Wistar rats (250–300 g; Charles River, Montreal, Canada) were deeply anesthetized using 4% isoflurane, and were sedated for the remainder of the surgery under 2% isoflurane. Animals received a two-level laminectomy of mid-thoracic vertebral segments T6–T7. A modified clip calibrated to a closing force of 35 g was applied extradurally to the cord for 1 minute and then removed. The animals were divided into four groups in a randomized and “blinded” manner, (1) Sham control group (laminectomy only – no SCI), (2) Non-injected injured control group (laminectomy and SCI – no injection), (3) AdV -ZFP-VEGF treatment group, and (4) AdV-eGFP control group. Using a stereotaxic frame and glass capillary needle (tip diameter 60 µm) connected to a Hamilton microsyringe, a total of 5×10^8^ viral plaque forming units (PFU) were injected into the dorsal spinal cord 24 hours post-SCI. Four 2.5 µl (10 µl total) intraspinal injections were made bilaterally at 2 mm rostral and caudal of the injury site. The injection rate is 0.60 µl/min and when the injection was completed, the capillary needle was left in the cord for at least 1 min to allow diffusion of the virus from the injection site and to prevent back-flow. The incision was closed in layers using standard silk sutures and animals were given a single dose of buprenorphine (0.05 mg/kg). Animals were allowed to recover in their cage under a heat-lamp and, subsequently, were housed in a temperature-controlled warm room (26°C) with free access to food and water. Animals were given buprenorphine (0.05 mg/kg) every 12 hours for 48 hours following surgery, and their bladders were manually voided three times daily. A subcutaneous injection of 10 mg/kg of cyclosporin A was administered daily starting 24 hours prior to the SCI until the end of the experiments for immunosuppression.

### Western Blotting

Following deep inhalation anesthetic, animals (n = 4–5/group) were sacrificed at five or ten days post-SCI and a 5 mm length of the spinal cord centered at the injury site was extracted. Samples were mechanically homogenized in 400 µl of homogenization buffer (0.1 M Tris, 0.5 M EDTA, 0.1% SDS, 1 M DTT solution, 100 mM PMSF, 1.7 mg/ml aprotinin, 1 mM pepstatin, 10 mM leupeptin) and centrifuged at 15,000 rpm for 10 minutes at 4°C. Supernatants were extracted and used for western blot analysis, where 20 µg of protein was loaded into 7.5% or 12% polyacrylamide gels (Bio-Rad, Mississauga, Canada). Membranes were probed with either monoclonal anti-NF200 antibody (1∶2000; Sigma, Oakville, Canada), rabbit IgG anti-VEGF-A antibody (1∶100; Santa Cruz Biotechnology, Santa Cruz, CA), or rabbit IgG anti-NFκBp65 (1∶1000; Santa Cruz Biotechnology, Santa Cruz, CA). NFκBp65 rabbit polyclonal antibody was used to recognize the p65 activation domain in the ZFP-VEGF treated animals. Primary antibodies were labelled with horseradish peroxidase-conjugated secondary antibodies (goat anti-mouse/rabbit IgG, 1∶3000; Jackson Immuno Research Laboratories, West Grove, PA), and bands were imaged using an enhanced chemiluminescence (ECL) detection system (Perkin Elmer, Woodbridge, Canada). Mouse monoclonal, beta-actin (Chemicon International, Inc., Temecula, CA) was immunoblotted as a loading control. Quality One detection software (Bio-Rad Laboratories, Hercules, CA) was used for integrated optical density (OD) analysis.

### Histochemistry

#### Histological Processing

Five or 10 days (n = 4–5/group), or 8 weeks (n = 10/group) post-SCI, following deep inhalation anesthetic, animals were transcardially perfused with 4% paraformaldehyde (PFA) in 0.1 M PBS. Then, the tissues were cryoprotected in 20% sucrose in PBS. A 10 mm length of the spinal cord centered at the injury site was fixed in tissue-embedding medium. The tissue segment was snap frozen on dry ice and sectioned on a cryostat at a thickness of 14 µm. Serial spinal cord sections at 500 µm intervals were stained with myelin-selective pigment luxol fast blue (LFB) and the cellular stain hematoxylin-eosin (HE) to identify the injury epicenter. Tissue sections showing the largest cystic cavity and greatest demyelination were taken to represent the injury epicenter.

#### Immunohistochemistry

The following primary antibodies were used: mouse anti-NeuN (1∶500; Chemicon International, Inc., Temecula, CA) for neurons, mouse anti-GFAP (1∶500; Chemicon International, Inc., Temecula, CA) for astrocytes, mouse anti-APC (CC1, 1∶100; Calbiochem, San Diego, CA) for oligodendrocytes, and mouse anti-RECA-1 (1∶25; Serotec Inc., Raleigh, NC) for endothelial cells. The sections were rinsed three times in PBS after primary antibody incubation and incubated with either fluorescent Alexa 568, 647 or 488 goat anti-mouse/rabbit secondary antibody (1∶400; Invitrogen, Burlington, Canada) for 1 hour. The sections were rinsed three times with PBS and cover slipped with Mowiol mounting medium containing DAPI (Vector Laboratories, Inc., Burlingame, CA) to counterstain the nuclei. The images were taken using a Zeiss 510 laser confocal microscope.

#### Quantification of Blood Vessels

Tissue sections – taken from animals sacrificed 10 days post-SCI – were used for immunofluorescence studies with a monoclonal antibody specific for RECA-1 (Rat Endothelial Cell Antibody). Vessels counts were performed on 4 selected fields (ventral horn, dorsal horn, left and right lateral columns) in each section under 25X magnification (0.14 mm^2^). The number of RECA-1-positive vessels was calculated at 2 mm and 4 mm, both rostral and caudal from the epicenter, for each animal.

#### Quantification of Angiogenesis

Tissue sections – taken from animals sacrificed 5 days post-SCI – were used to quantify angiogenesis following SCI and AdV-ZFP-VEGF administration. Angiogenesis was calculated as vessels co-labelled with RECA-1 and Ki67 (cellular proliferation). Angiogenesis quantification was performed on 4 selected fields (ventral horn, dorsal horn, left and right lateral columns) in each section under 25X magnification (0.14 mm^2^). The number of angiogenic vessels was calculated at 1, 2 and 3 mm, both rostral and caudal from the epicenter, for each animal. Rostral and caudal values were pooled for each distance.

#### Quantification of Apoptosis

An *in situ* terminal-deoxy-transferase mediated dUTP nick end-labeling (TUNEL) apoptosis kit (Chemicon International, Inc., Temecula, CA) was used to label apoptotic cells in tissues extracted from animals 5 days post-SCI. TUNEL staining was completed as described in the manufacturer's instructions. The numbers of TUNEL positive nuclei were counted at the epicenter, as well as at 1, 2 and 3 mm (rostral and caudal) from the injury epicenter. In each tissue section, the whole section was counted to include all apoptotic nuclei visible.

#### Quantification of Neurons

Tissue sections – taken from animals sacrificed 5 days post-SCI – were used for immunofluorescence studies with a monoclonal antibody specific for NeuN (Neuronal Nuclei). Neuron quantification was conducted only in the grey matter under 25X magnification (0.14 mm^2^), and all cells were counted. The number of NeuN-positive cells was calculated at 1, 2 and 3 mm, both rostral and caudal from the epicenter, as well as at the epicenter.

#### Assessment of Tissue Sparing and Cavity Formation

Tissue sparing and cavity formation was analyzed 8 weeks after SCI, at the center of the lesion, 2 mm above and 2 mm below the epicenter. Sections were stained with LFB-HE. The measurements were carried out on coded slides using StereoInvestigator® software (MBF Bioscience, Williston, VT). Cross-sectional residual tissue and cavity areas were normalized with respect to total cross-sectional area and the areas were calculated every 500 µm within the rostrocaudal boundaries of the injury site.

### Behavioural Testing

#### Open-field Locomotor Scoring

Locomotor recovery of the animals (n = 8–10/group) was assessed by two independent observers using the 21 point Basso, Beattie, and Bresnahan (BBB) open field locomotor score [15] from 1 to 8 weeks after SCI. The BBB scale was used to assess hindlimb locomotor recovery including joint movements, stepping ability, coordination, and trunk stability. Testing was done every week on a blinded basis and the duration of each session was 4 min per rat. Scores were averaged across both the right and left hindlimbs to arrive at a final motor recovery score for each week of testing.

#### Automated Gait Analysis (CatWalk)

Gait analysis was performed using the CatWalk system (Noldus Information Technology, Wageningen, Netherlands) as described [16], [17]. In short, the system consists of a horizontal glass plate and video capturing equipment placed underneath and connected to a PC. In our work, for correct analysis of the gait adaptations to the chronic compression, after standardization of the crossing speed, the following criteria concerning walkway crossing were used: (1) the rat needed to cross the walkway, without any interruption (2) a minimum of three correct crossings per animal were required. Files were collected and analyzed using the CatWalk program, version 7.1. Individual digital prints were manually labeled by one observer blinded to groups. With the CatWalk, a vast variety of static and dynamic gait parameters can be measured during spontaneous locomotion. In the present study, a blinded examiner generated and analyzed data for the following parameters:

- forelimb stride length (expressed in mm): distance between two consecutive forelimb paw placements
- hindlimb print area – maximal area of the paw print in contact with the detection surface of the CatWalk™ (expressed in mm^2^)
- hindlimb print width – the maximal distance spanning the medial and lateral contact points of the paw (expressed in mm)
- hindlimb print length – the maximal distance spanning the cranial and caudal contact points of the paw (expressed in mm)
- hindlimb swing speed (expressed in pixels/sec): is the speed of the paw during the swing phase (the duration of no paw contact with the glass plate during a step cycle).

Acclimation and training to the walking apparatus were performed as described by Gensel *et al.* [18]. Since Catwalk quantifies weight support and stepping, only a sub-set of animals exhibiting weight support (BBB scores >9) were used in the CatWalk experiments (n = 5/group). Most AdV-eGFP animals did not reach BBB scores >9; however, animals for this group were subject to CatWalk™ analysis to provide consistency in our experiments.

#### Mechanical Allodynia

At-level mechanical allodynia was determined at 4 weeks and 8 weeks post-SCI using 2 g and 4 g von Frey monofilaments as previously described [19]. Animals were acclimatized for 30 minutes in an isolated room for 30 minutes prior to pain testing. The von Frey monofilament was applied to the dorsal skin surrounding the incision/injury site 10 times and animals' behavioural response to each was recorded. An adverse response to the application of the monofilament (determined in advance of experiments) included vocalization, licking, biting and immediate movement to the other side of the cage. The proportion of rats to exhibit allodynia in each group is reported, and an increased number of responses was associated with the development of at-level mechanical allodynia. Below-level mechanical allodynia was determined by quantifying the pain threshold of the hindpaws. Animals were placed in stance on a raised grid, allowing von Frey filaments to be applied to the plantar surface of the hindpaw. Increasing monofilaments were used (2, 4, 8, 10, 16, 21, and 26 g) until the animal displayed an adverse response (as described above). The weight of the von Frey filament that elicited the response was recorded as the pain threshold value, with lower threshold values indicating increased sensitivity to mechanical stimuli (and perhaps the development of mechanical allodynia). Finally, below-level thermal allodynia was assessed using the tail flick method. A 50°C thermal stimulus was applied to the distal portion of the animals' tail by a Tail Flick Analgesia Meter (IITC Inc. Life Science, Woodland Hills, California, USA), and the time for the animal to remove its tail from the stimulus was recorded. The latency time is graphed for each treatment group, and decreased latency times were associated with the development of thermal allodynia.

### Electrophysiology

#### Motor Evoked Potentials

Motor evoked potential recordings (MEPs): In addition to the behavioural assessements, MEPs were recorded *in vivo* to assess the physiological integrity of spinal cord. This approach has been extensively used in our laboratory in rodent models of SCI. *In vivo* recordings of motor evoked potentials were recorded from the each of the treatment and control groups at 8 weeks post-injury (n = 6/group). For MEPs, rats were under light isoflurane anaesthesia (<1%), and recordings were obtained from hindlimb biceps femoris muscle. Stainless steel subdermal needle electrodes were inserted into the muscle. Recordings were acquired using Keypoint Portable (Dantec Biomed, Denmark). A reference electrode was placed under the skin between the recording and stimulating electrodes. Stimulation was applied to the midline of the cervical spinal cord using a silver ball electrode (0.13 Hz; 0.1 ms; 2 mA; 200 sweeps). The interlaminar ligaments were removed and a small amount of bone was removed from the vertebra (not a full laminectomy, just enough to create a space for the electrode to reach the cervical cord). The amplitude was determined by the difference between the positive peak and negative peak. Latency was calculated as the time from the start of the stimulus artifact to the first prominent peak. For individual rats, the average of peak amplitude and latency was averaged from 200 sweeps and analyses were undertaken by ANOVA.

#### H-Reflex

The Hoffmann reflex is one of the most studied reflexes in humans and is the electrical analogue of the monosynaptic stretch reflex. The H-reflex is evoked is evoked by low-intensity electrical stimulation of the afferent nerve, rather than a mechanical stretch of the muscle spindle, that results in monosynaptic excitation of alpha-motorneurons. H-reflex can be used as a tool (in combination with other outcome measures) to examine spasticity and short- and long-term plasticity. Recording electrodes were placed two centimeters apart in the mid-calf region and the posterior tibial nerve was stimulated in the popliteal fossa using a 0.1 ms duration square wave pulse at a frequency of 1 Hz. The rats were tested for maximal plantar H-reflex/maximal plantar M-response (H/M) ratios to determine the excitability of the reflex. The recordings were filtered between 10–10000 Hz.

### Statistical Analysis

Data were analyzed with SigmaPlot software (Systat Software Inc., San Jose, California, USA). For data that investigated the percentage of cells, the data were subject to an arcsine transformation prior to statistical analysis to attain a more normal distribution. For comparison of groups sampled at various distances from the injury site (TUNEL, RECA-1, NeuN), a two-way analysis of variance (ANOVA) with repeated measures was used, followed by the post-hoc Holm-Sidak test. For comparisons of multiple groups at a single time point (Western blotting, BBB, Catwalk, Electrophysiology), a one-way ANOVA was performed, followed by the post-hoc Holm-Sidak test.

The Holm-Sidak post-hoc was used, as it is recommended as the best multiple comparisons test following an ANOVA [20], [21]. The Holm-Sidak test is more sensitive and powerful compared to Bonferroni or Tukey post-hoc tests, therefore it is more likely to detect all significant results and increases the probability of not committing type II errors (reduces the chance of rejecting something that is true).

In all figures, the mean value ± SEM are used to describe the results. Statistical significance was accepted for p values of <0.05.

## Results

### AdV-ZFP-VEGF Delivery into the Injured Spinal Cord

**Figure 1:**
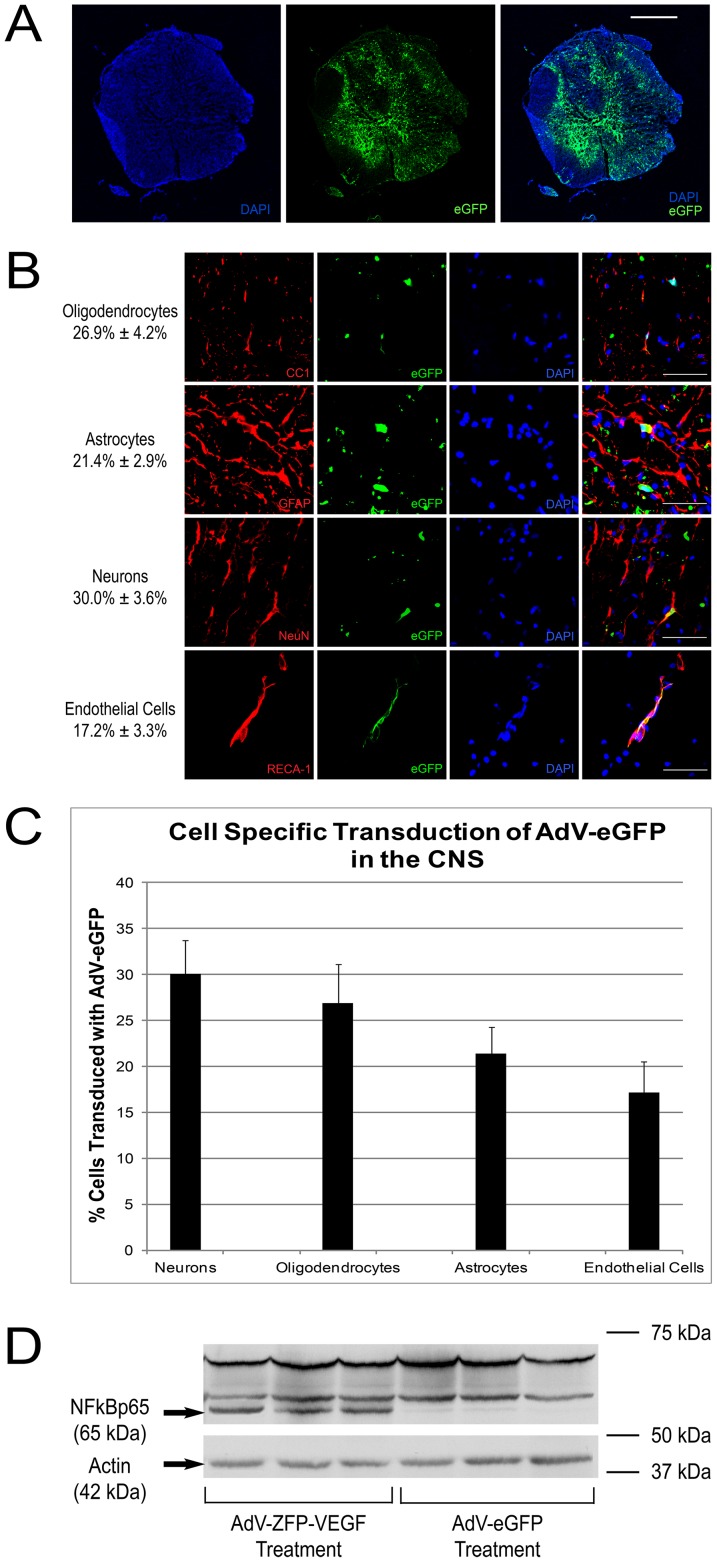
Transduction of AdV-eGFP/AdV-ZFP-VEGF into the spinal cord. (A) Photomicrographs showing a transverse section of rat spinal cord obtained adjacent to the injury site 10 days after spinal cord injury and AdV-eGFP injection. eGFP signal was detected in both the gray matter and white matter. (B) High-power (63X) confocal images show that the AdV-eGFP vector (green) transfected neurons (NeuN), astrocytes (GFAP), oligodendrocytes (CC1) and endothelial cells (RECA-1). Cells have been counter-stained with DAPI (blue) as nuclear marker. (C) Bar graph displays quantification of transduced cell types ± SEM, as identified by the cell-specific markers NeuN, GFAP, RECA-1 and CC1. (D) Evaluation of AdV-ZFP-VEGF gene transfer. Western blot showed that the NFκB p65 rabbit polyclonal antibody recognizes the p65 activation domain in the AdV-ZFP-VEGF treated animals. The higher molecular weight bands are endogenous NFκBp65 fragments, which are also recognized by the antibody; however, these bands are present in both the control and treatment groups. The lower band (arrow) corresponds to the AdV-ZFP-VEGF and was only present in the treated animals. Lower panel shows actin expression as a protein control. Scale bar: 1000 µm for A; 100 µm for B.

To evaluate the transduction efficiency of the adenoviral constructs *in vivo*, AdV-eGFP was injected into animals at 24 hours post-SCI. The AdV-eGFP fluorescent signal was detected in both the white and grey matter of the injured spinal cord five days after SCI (Figure 1A). Figures 1B and 1C demonstrate eGFP expression in neurons, astrocytes, endothelial cells and oligodendrocytes, indicating successful adenoviral transduction into each cell type. Further quantification of co-labelled cells showed that AdV vector non-preferentially transduces all cell types (Neurons – 30.0%±3.6%, Oligodendrocytes – 26.9%±4.2%, Astrocytes – 21.4%±2.9%, Endothelial cells – 17.2%±3.3%). Since the AdV-ZFP-VEGF construct contains the p65 subunit of the human NFκB transcription factor as the activation domain [12], we were able to confirm delivery of AdV-ZFP-VEGF by immunoblotting using an NFκB p65 antibody to detect the presence of the transcription factor (Figure 1D). As a positive control, HEK293 cells were transduced with ZFP-VEGF and cell lysates were processed for immunoblotting using the same NFκB p65 antibody (data not shown). These results demonstrate the successful delivery of a localized gene therapy to the injured spinal cord.

### VEGF mRNA and protein expression is increased following 24 hour delayed AdV-ZFP-VEGF administration

**Figure 2:**
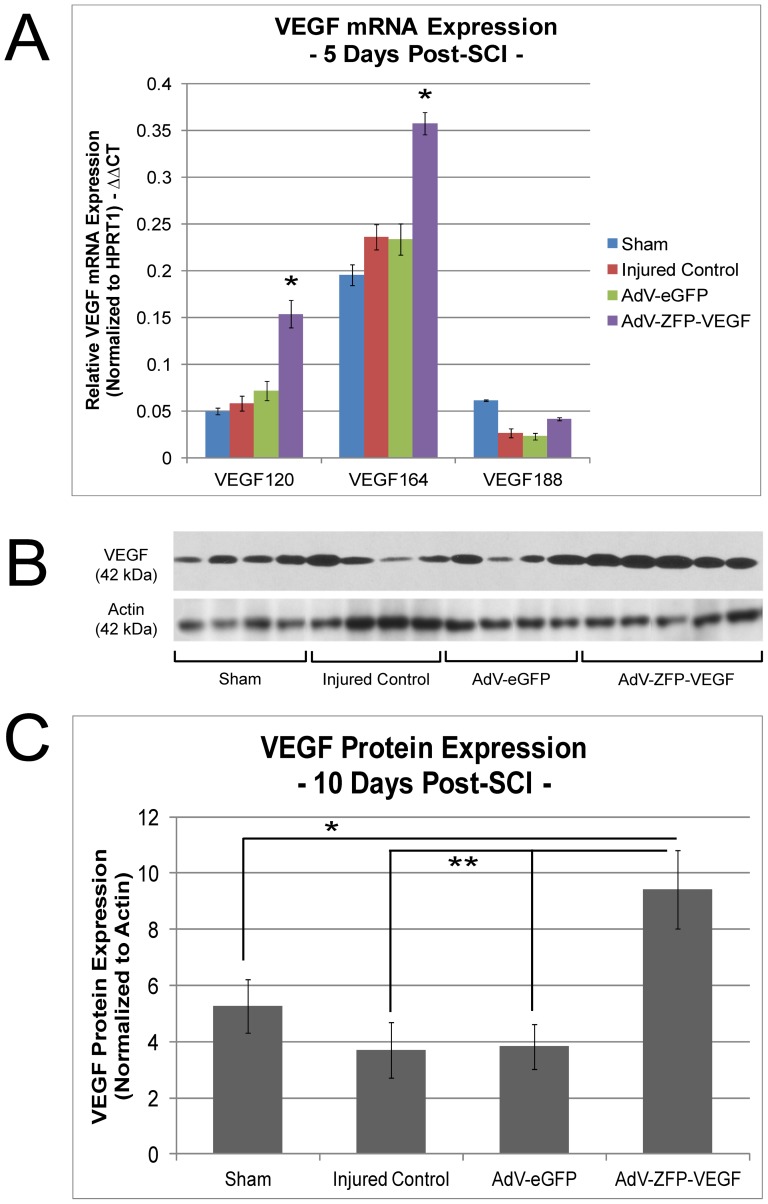
AdV-ZFP-VEGF increases VEGF mRNA and protein. (A) VEGF mRNA levels encoding for VEGF_120_, VEGF_164_ and VEGF_188_ isoforms were measured by quantitative real-time PCR at 5 days post-SCI. The bar graph illustrates that administration of ZFP-VEGF resulted in an increase of VEGF mRNA compared with AdV-eGFP and SCI injured control groups. Relative mRNA levels are expressed as the mean ± SEM, n = 4/sham and injured control groups, n = 5/AdV-eGFP and AdV-ZFP-VEGF groups. One-way ANOVA (Holm-Sidak post-hoc) was completed individually for each isoform **p<0.001, *p<0.01. (B) Western blot showing administration of AdV-ZFP-VEGF resulted in increased VEGF-A protein levels at 10 days post-SCI, and (C) Quantification shows a significant increase in VEGF-A 42 kD protein in AdV-ZFP-VEGF treated animals compared with control groups. Optical density (OD) of VEGF-A was normalized to actin. Data are presented as mean ± SEM, n = 4/sham, injured control and AdV-eGFP treated groups and n = 5/AdV-ZFP-VEGF treated group. One-way ANOVA (Holm-Sidak post-hoc) **p<0.02, *p<0.05.

**Figure 3:**
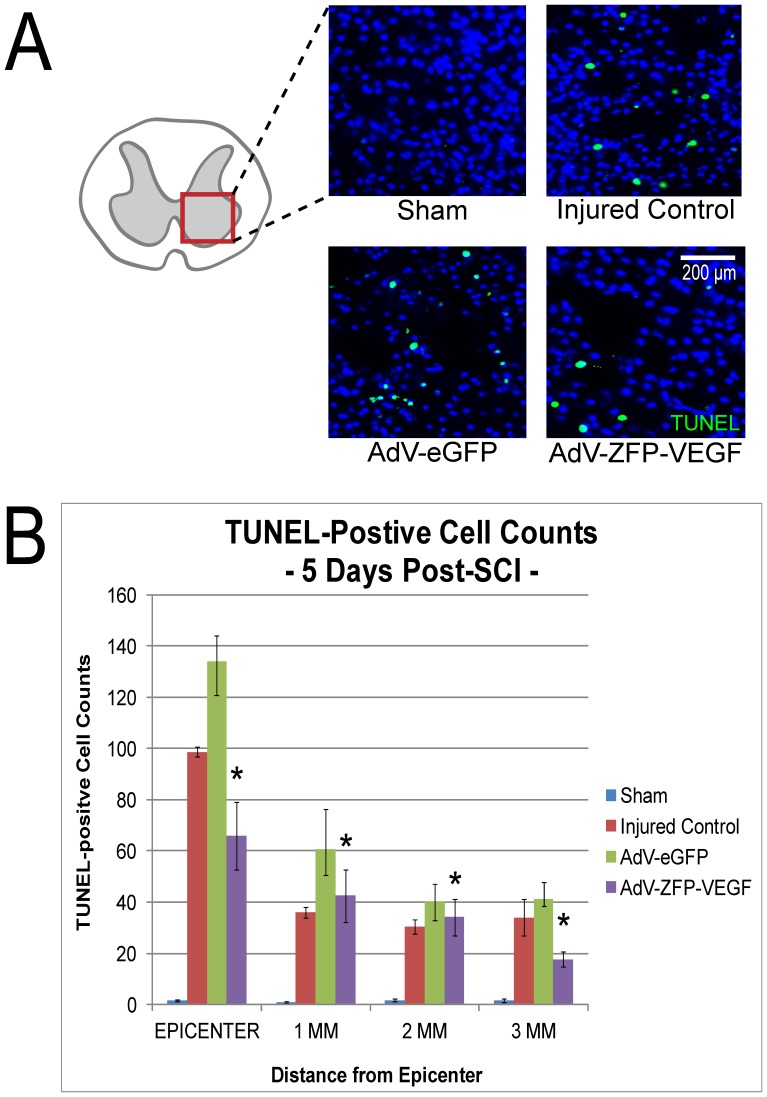
AdV-ZFP-VEGF administration reduces apoptosis after SCI. (A) Representative sections taken 2 mm rostral to the epicenter from animals sacrificed at 5 days post-SCI and tissue processed with TUNEL staining (green); scale 200 µm. An overall reduction of TUNEL-positive cells was observed in the AdV-ZFP-VEGF treated group. Cells have been counter-stained with DAPI (blue) as nuclear marker (B) Bar graph shows quantification of the TUNEL-positive cell counts at 5 days after SCI (pooled values from rostral and caudal counts). There was a significant decrease in TUNEL-positive cells in the AdV-ZFP-VEGF treatment group versus all other injured groups (when compared against other groups and all distances). Values are mean ± SEM, n = 4/sham and injured control groups, n = 5/AdV-eGFP and AdV-ZFP-VEGF groups. Two-way ANOVA (Holm-Sidak post-hoc), *p<0.01.

Animals were sacrificed 5 days post-SCI and mRNA expression levels of three predominant VEGF isoforms found in the CNS – VEGF_120_, VEGF_164_ and VEGF_188_ – were measured by quantitative real-time PCR (qRT-PCR). Figure 2A shows that 24 hour delayed administration of AdV-ZFP-VEGF resulted in significant increases in VEGF mRNA of isoforms 120 (p<0.001), 164 (p<0.001), but not isoform 188, when compared with AdV-eGFP control animals and injured control animals (n = 4/sham and injured control; n = 5/AdV-ZFP-VEGF and AdV-eGFP). VEGF-A protein expression was assessed at 10 days following SCI by Western blot using anti-VEGF antibodies, which detect the 42 kDa and 21 kDa bands and are recommended for the detection of the 189, 165 and 121 amino acid splice variants of VEGF. In Figures 2B and 3C, we show that the 42 kDa VEGF-dimer protein was significantly increased by approximately 2.5-fold in AdV-ZFP-VEGF treated animals versus AdV-eGFP and injured control groups (p<0.02), and by approximately 1.8-fold in AdV-ZFP-VEGF treated animals compared to sham animals (p<0.05) (n = 4/sham, injured control and AdV-eGFP, n = 5/AdV-ZFP-VEGF group). Previous studies using AdV-ZFP-VEGF have shown increases in VEGF mRNA and protein levels [11], [22]–[24]. Consistent with these studies, our results confirm that AdV-ZFP-VEGF increases both mRNA and protein levels of VEGF in the spinal cord following 24 hour delayed administration.

### Apoptosis is reduced in animals treated with AdV-ZFP-VEGF 24 hours post-SCI

Our laboratory has previously shown that apoptotic cell death occurs as early as 6 hours following SCI and persists until 14 days post injury [25]. To assess the effects of AdV-ZFP-VEGF treatment on apoptotic cell death, *in situ* terminal-deoxy-transferase mediated dUTP nick end-labeling (TUNEL) staining was performed 5 days after injury (Figure 3). TUNEL-positive cells were found evenly distributed through the gray and white matter in the injured spinal cord, with the greatest apoptosis observed near the injury epicenter. TUNEL-stained nuclei were counted at the injury epicenter, and at 1, 2, and 3 mm from the injury epicenter both rostral and caudal to the lesion site, but rostral and caudal values were pooled. Figure 3B shows that AdV-ZFP-VEGF treatment was associated with an overall significant reduction in the number of TUNEL-positive cells rostral and caudal from the injury epicenter, when conducting a two-way ANOVA for distance from the injury epicenter and treatment group (Two-way ANOVA, Holm-Sidak post-hoc; p<0.01; n = 4/sham and injured control groups, n = 5/AdV-eGFP and AdV-ZFP-VEGF groups).

### 24 hour delayed AdV-ZFP-VEGF administration provides neuroprotection

**Figure 4:**
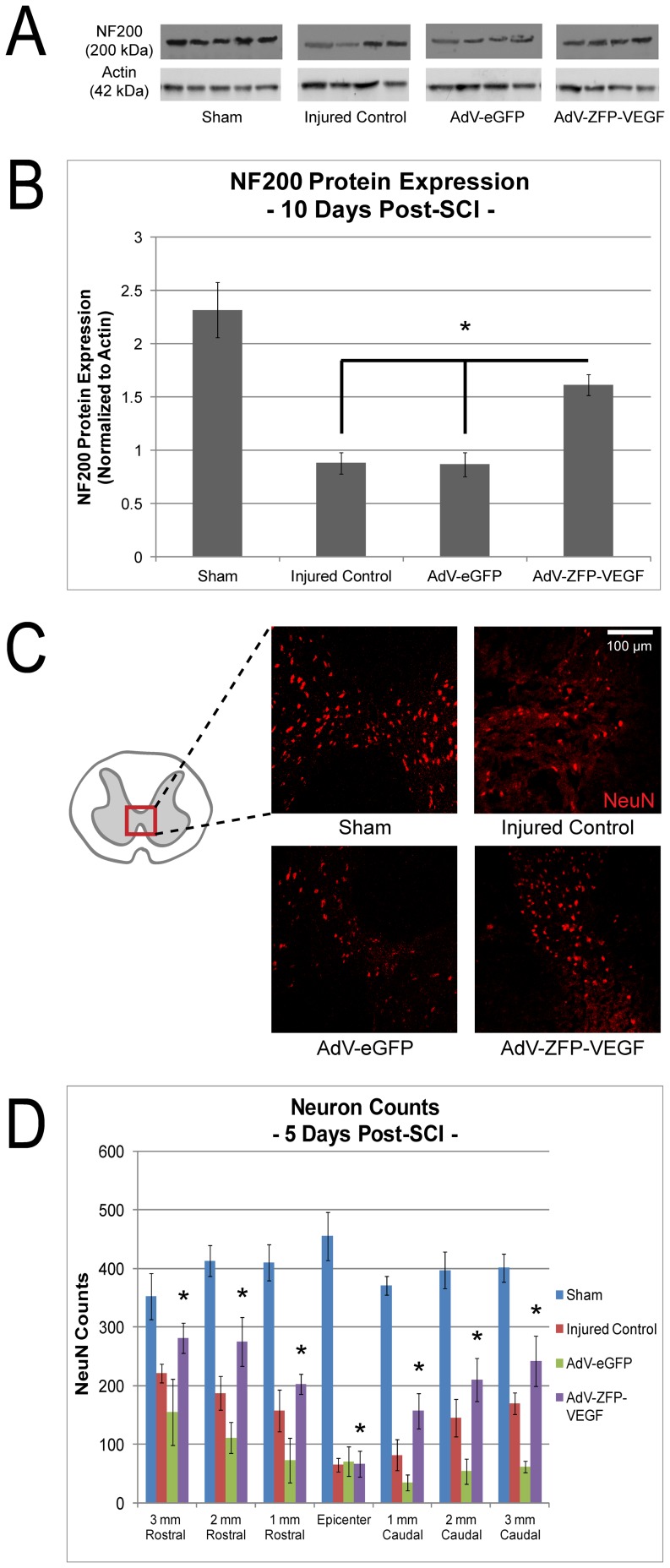
AdV-ZFP-VEGF administration attenuated axonal degradation and increased neuron sparing. (A) Western blot indicates that administration of AdV-ZFP-VEGF resulted in a significant attenuation of NF200 degradation 10 days after injury. Lower panel shows actin protein control. (B) Relative OD value of controls versus AdV-ZFP-VEGF treated animals. Significant NF200 sparing was observed in AdV-ZFP-VEGF-treated animals compared to control groups at 10 days after injury, although all injured groups showed significant NF200 loss following SCI. Optical density of NF200 was normalized to actin. One-way ANOVA (Holm-Sidak post-hoc), *p<0.05. (C) Representative sections taken 2 mm rostral to the epicenter from AdV-ZFP-VEGF treated and AdV-eGFP treated animals immunostained with NeuN at 5 days after SCI; scale 200 µm. A greater number of NeuN-positive cells were observed in animals treated with AdV-ZFP-VEGF. (D) Bar graph shows quantification of the NeuN-positive cell counts at 5 days after SCI. There was a significant preservation of neurons overall in the AdV-ZFP-VEGF group compared to all the other injured groups (two-way ANOVA comparing distance from the epicenter and treatment group). Bar graph shows mean OD values ± SEM. Two-way ANOVA (Holm-Sidak post-hoc), *p<0.02. n = 5/sham, n = 4/injured control, AdV-eGFP and AdV-ZFP-VEGF groups.

Neurofilament protein (NF200), a hallmark protein lost following neurodegeneration, was quantified in the injured region of the cord to assess the neuroprotective effects of AdV-ZFP-VEGF after SCI. Previous research from our laboratory indicated a significant loss of NF200 after SCI [26], [27]. As shown in Figure 4, the amount of NF200 protein was significantly increased by approximately 2-fold at 10 days following SCI in animals treated with AdV-ZFP-VEGF versus control animals (Figure 4A and 4B) (p<0.05).

To further assess the neuroprotective effects of AdV-ZFP-VEGF following SCI, we quantified spared neurons 5 days after injury. NeuN, which recognizes neuronal cell bodies, was used to identify neurons in cross-sections of spinal cord tissue. Figures 4C and 4D demonstrate that AdV-ZFP-VEGF treatment results in a significant sparing of neurons spanning the lesion site, when compared to injured control and AdV-eGFP animals (Two-way ANOVA comparing AdV-ZFP-VEGF with other injured animals across all distances from the epicenter, Holm-Sidak post-hoc; p<0.02). AdV-eGFP animals show a significant decrease in NeuN counts compared to injured control animals (p<0.01), and the additional loss of cells is likely attributed to the physical damage caused by the intraspinal injections.

### 24 hour delayed AdV-ZFP-VEGF administration results in an increased number of vessels and promotes angiogenesis

**Figure 5:**
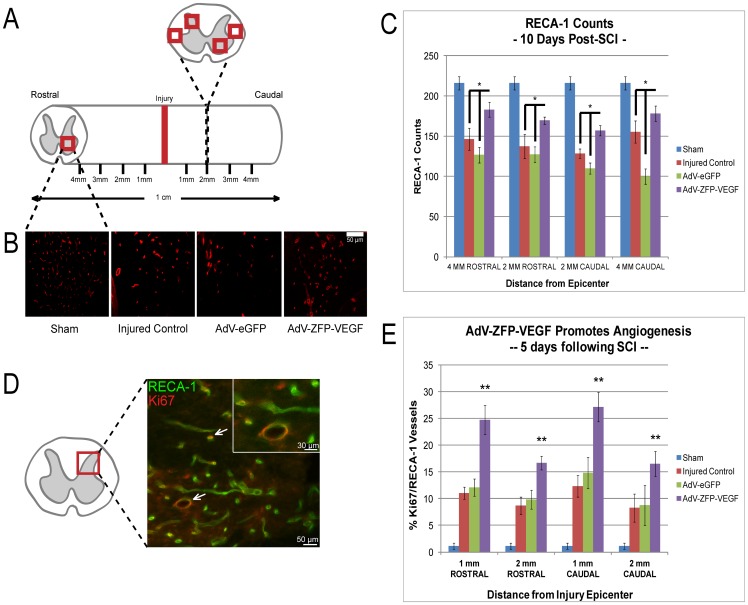
AdV-ZFP-VEGF results in increased vessel counts and angiogenesis. (A) Left panel: Illustration of the area of spinal cord areas used for RECA-1 counting (2 grey matter areas, 2 white matter areas). (B) Representative sections taken 2 mm rostral to the epicenter from a AdV-ZFP-VEGF treated and AdV-eGFP control animal respectively immunostained with RECA-1 at 10 days after SCI; scale 100 µm. An increased number of vessels were observed in the AdV-ZFP-VEGF treated group. (C) Bar graph illustrating the RECA-1 positive cell counts 10 days after SCI. AdV-ZFP-VEGF administration resulted in a significant increase in vascular counts (2 mm and 4 mm away from the epicenter) as compared with the control group. (D) Representative confocal image from an ADV-ZFP-VEGF treated animal at 5 days post-injury. Image was taken at 2 mm rostral from the epicenter, and shows double-labeled cells. Cells were stained for endothelial cells (RECA-1, green) and proliferation (Ki67, red). Scale bar  = 50 µm (30 µm for magnified panel). (E) Angiogenesis was assessed by quantifying Ki67/RECA-1 co-labeled vessels. Data is presented at the percentage of RECA-1+ vessels that were also Ki67+, with an overall average increase of 10% vascular proliferation observed in the animals receiving AdV-ZFP-VEGF administration. All data are presented as mean ± SEM, and was analyzed by Two-way ANOVA (Holm-Sidak post-hoc). Angiogenesis data were analyzed by performing an arcsine transformation of the values, prior to Two-way ANOVA and post-hoc testing. *p<0.01, **p<0.001. n = 4/sham and injured control groups, n = 5/AdV-eGFP and AdV-ZFP-VEGF groups.

In order to quantify the vascular response to ZFP-VEGF, we conducted immunostaining with RECA-1, a monoclonal antibody specific for endothelial cells, at 10 days following SCI. The severity of the compression injury resulted in considerable disruption to the spinal cord vasculature at the injury epicentre, thus we were unable to quantify the epicenter accurately. Therefore, we assessed spinal cord tissue sections at 2 mm and 4 mm – both caudal and rostral – from the lesion epicenter (Figure 5A). Figures 5B and 5C show that AdV-ZFP-VEGF administration markedly increases the number of RECA-1-positive vessels both rostral and caudal, when compared to control animals (p<0.01). These results are consistent with previous findings from our laboratory in studies administering AdV-ZFP-VEGF immediately following injury [11].

To investigate some of the potential mechanisms of AdV-ZFP-VEGF action, we examined the effects of 24 hour delayed AdV-ZFP-VEGF administration on endothelial cell proliferation. One of the most characterized roles of VEGF is promoting angiogenesis in both embryonic development and wound healing [28], therefore we aimed to study if AdV-ZFP-VEGF administration would further promote angiogenesis. Tissues co-labeled with RECA-1 and Ki67 at 5 days following SCI indicated that AdV-ZFP-VEGF administration increased angiogenesis by approximately 10% (p<0.001) (Figure 5D and 5E). These results indicate that AdV-ZFP-VEGF administration, which results in an increase in VEGF expression, ultimately promotes angiogenic pathways following SCI. Other research has suggested that VEGF administration results in angiogenesis; however, these studies simply show an increase in the number of vessels present. Here, we demonstrate that VEGF increases endothelial cell proliferation *in vivo* following SCI, and to our knowledge, this is the first study to use a delayed VEGF therapy and demonstrate an increase in vessels, that is likely attributable to angiogenesis.

### AdV-ZFP-VEGF results in functional improvement

**Figure 6:**
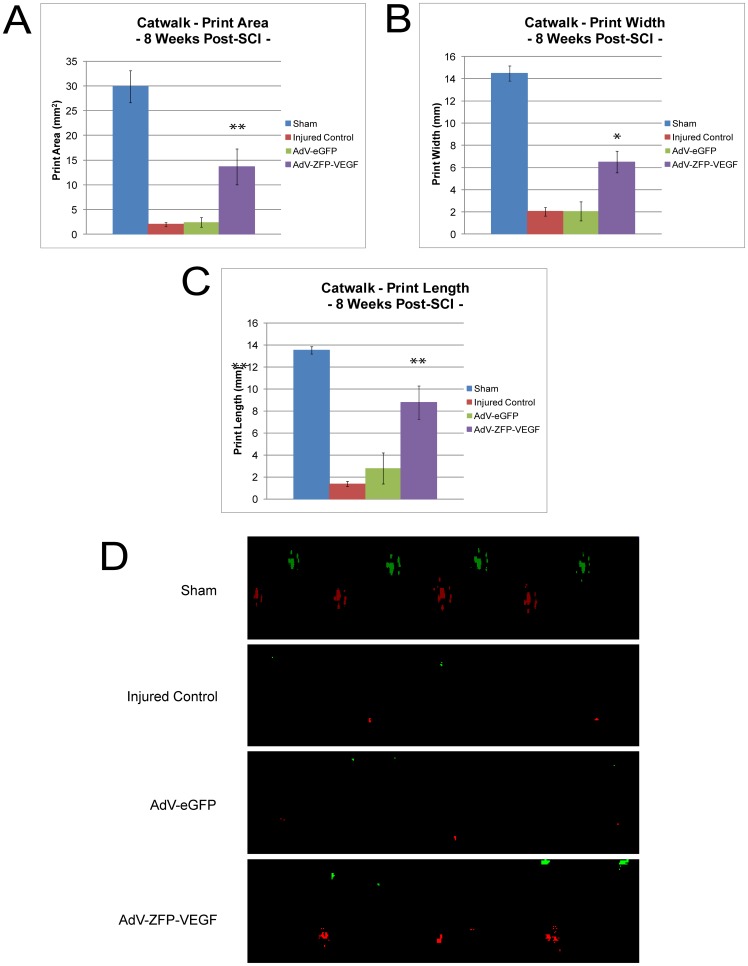
AdV-ZFP-VEGF improves hindlimb weight support. Catwalk gait analysis was used to assess hindlimb weight support. A sub-set of animals (with BBB scores >9) were assessed every week between 4–8 weeks, and each animal performed a standardized Catwalk run. A blinded observer analyzed the data. (A) Paw area: the maximal area of the paw print in contact with the detection surface of the CatWalk (expressed in mm^2^), (B) Paw width: the maximal distance spanning the medial and lateral contact points of the paw (expressed in mm), and (C) Paw length: the maximal distance spanning the cranial and caudal contact points of the paw (expressed in mm). (D) Representative images of CatWalk forelimb (green) and hindlimb (red) prints, which were used to quantify the data presented in Figure 6 and Figure 7. Data presented is the mean ± SEM, n = 5/group, at 8 weeks following SCI. One-way ANOVA (Holm-Sidak post-hoc). *p<0.05, **p<0.005.

**Figure 7:**
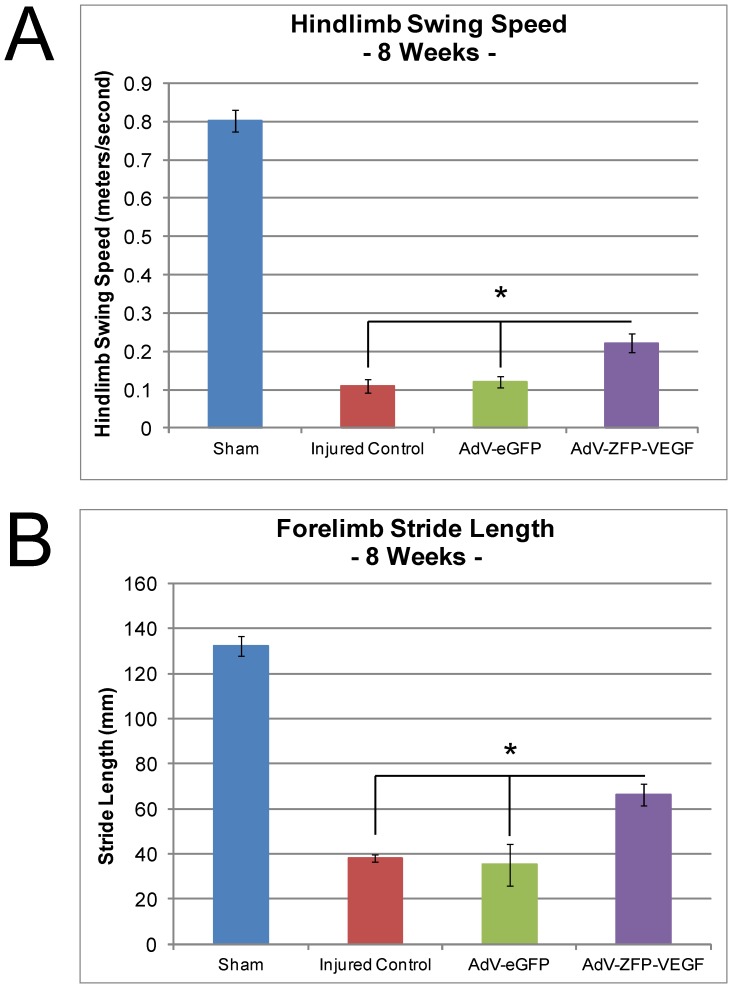
Forelimb and Hindlimb locomotion is improved by AdV-ZFP-VEGF administration. (A) Catwalk gait analysis was used to assess hindlimb swing speed. Animals were assessed every week between 4–8 weeks, and each animal performed a standardized Catwalk run. A blinded observer analyzed the data. Data presented is the mean ± SEM, n = 5/group, at 8 weeks following SCI. One-way ANOVA (Holm-Sidak post-hoc). *p<0.02. (B) Catwalk gait analysis was used to assess forelimb stride length. Animals were assessed every week between 4–8 weeks, and each animal performed a standardized Catwalk run. A blinded observer analyzed the data. Data presented is the mean ± SEM, n = 5/group, at 8 weeks following SCI. One-way ANOVA (Holm-Sidak post-hoc). *p<0.02.

At the cellular/molecular level, we have observed that AdV-ZFP-VEGF results in beneficial effects. However, in order to assess the viability of any therapy, these effects must be translated into functional gains. In our study, we assessed hindlimb function using open-field BBB scoring and Catwalk, between 1-8 weeks following SCI. Analysis of Catwalk data showed that animals treated with AdV-ZFP-VEGF had significantly improved hindlimb weight support (p<0.05) (Figure 6), hindlimb swing speed (p<0.02) (Figure 7), and forelimb stride length (p<0.02) (Figure 7) compared to all other injured control groups. Enhancements in hindlimb weight support and overall gait (hindlimb swing speed, and forelimb stride length) are important changes that may reflect an improved quality of life of individuals suffering with SCI.

### AdV-ZFP-VEGF does not result in improved BBB scores

**Figure 8:**
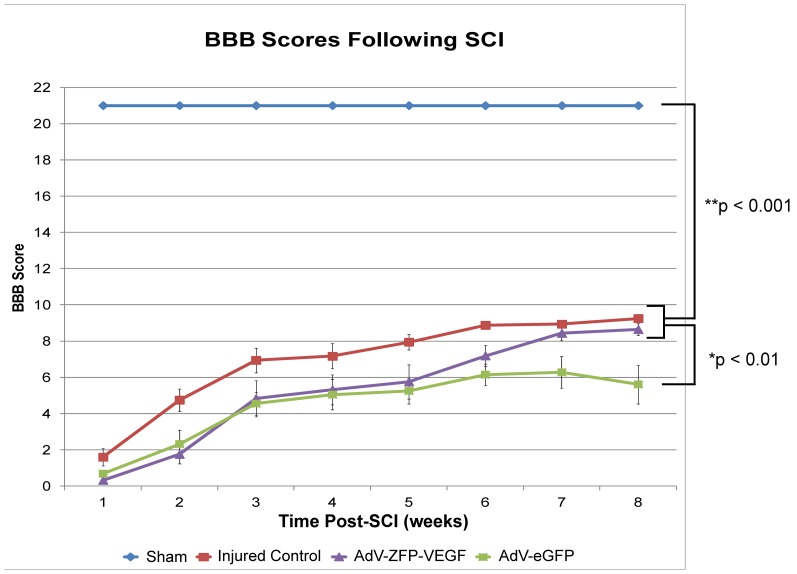
AdV-ZFP-VEGF does not improve open-field walking (BBB) scores following SCI. Open-field locomotion was assessed using the 21-point BBB scale. Animals were assessed weekly for 8 weeks following injury by blinded observers (n = 8/sham and AdV-ZFP-VEGF groups; n = 10/injured control and AdV-eGFP groups). The left and right limbs were scored individually, but the data presented is the average between left and right hindlimb recovery.

AdV-ZFP-VEGF treated animals did not show improved BBB scores, compared in injured control animals, although they did perform better than AdV-eGFP injected animals (p<0.01) (Figure 8). Significant recovery shown by CatWalk (animals analyzed were a sub-set of animals that achieved >8 for BBB scoring) do not correspond with significantly improved BBB scores between injured control and AdV-ZFP-VEGF animals at 8 weeks post-injury. In the discussion we will provide a more detailed explanation that may validate these findings; however, the discrepancy between BBB and CatWalk data may be due to the more qualitative nature of the BBB, as opposed to the quantitative gait analysis software.

### Delayed AdV-ZFP-VEGF administration does not improve motor evoked potentials or H-reflex following SCI

**Figure 9:**
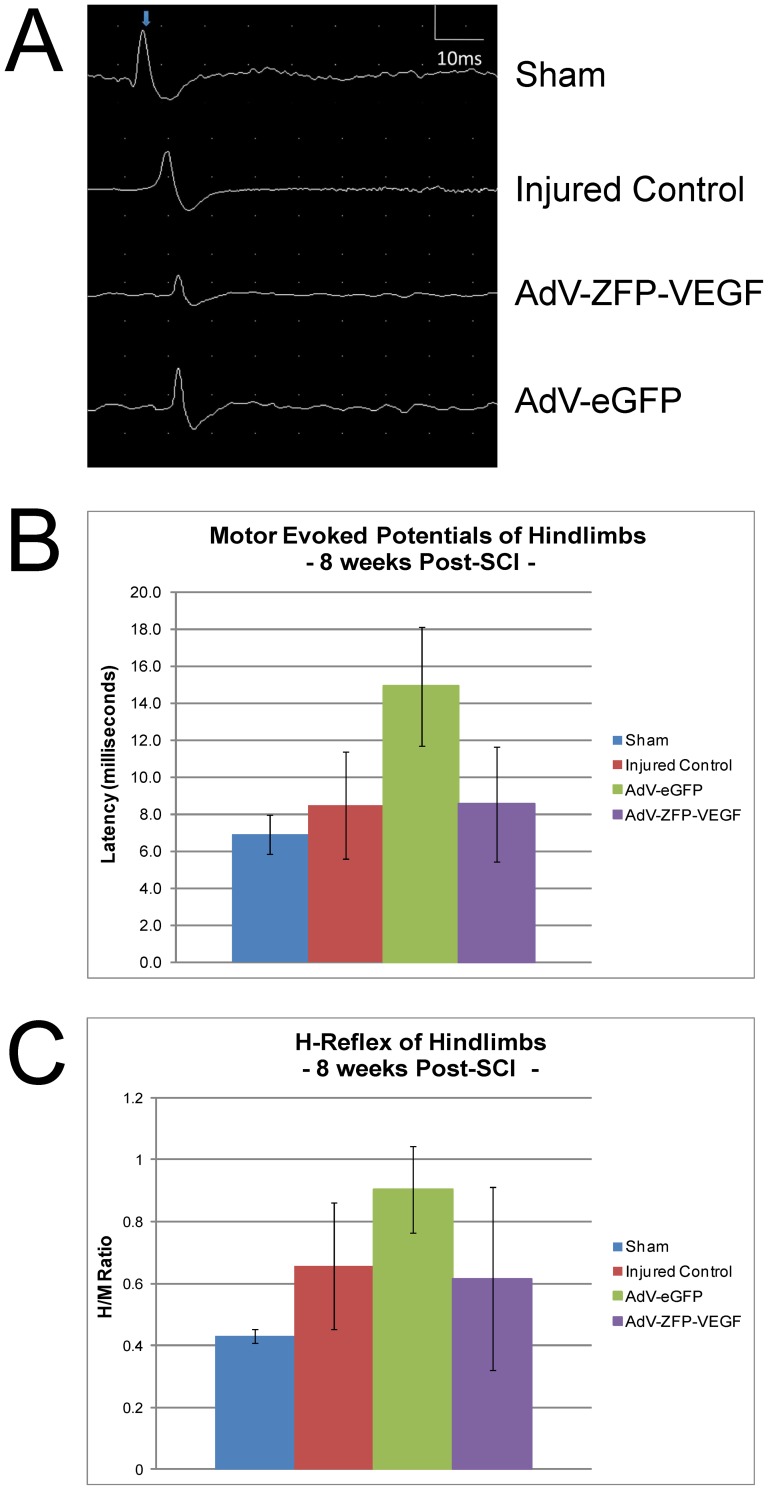
Electrophysiological assessment following AdV-ZFP-VEGF administration. (A) Representative tracings of MEP's recorded from the hindlimb at 8 weeks post-injury. (B) MEP quantification. Recordings were obtained from hindlimb biceps femoris. Stimulation was applied to the midline of the cervical spinal cord (0.13 Hz; 0.1 ms; 2 mA; 200 sweeps). Latency was calculated as the time from the start of the stimulus artifact to the first prominent peak. AdV-ZFP-VEGF did not result in improved MEP's. (C) H-Reflex quantification. Recording electrodes were placed two centimeters apart in the mid-calf region and the posterior tibial nerve was stimulated in the popliteal fossa using a 0.1 ms duration square wave pulse at a frequency of 1 Hz. The rats were tested for maximal plantar H-reflex/maximal plantar M-response (H/M) ratios to determine the excitability of the reflex. AdV-ZFP-VEGF administration did not significantly alter the H/M ratio. n = 6/group.

To further examine the functional changes we performed *in vivo* electrophysiology on the hindlimbs of animals at 8 weeks post-SCI. Our data indicate that although AdV-ZFP-VEGF treated animals show an improved gait via CatWalk™ analysis, we did not observe any significant improvements in axonal conduction in the hindlimbs, as assessed by motor evoked potential recordings (Figure 9A and 9B). We also examined the H-reflex (H/M ratios) following SCI as a measure of spasticity, and observed no electrophysiological differences between groups (Figure 9B).

### AdV-ZFP-VEGF administration significantly reduces allodynia

**Figure 10:**
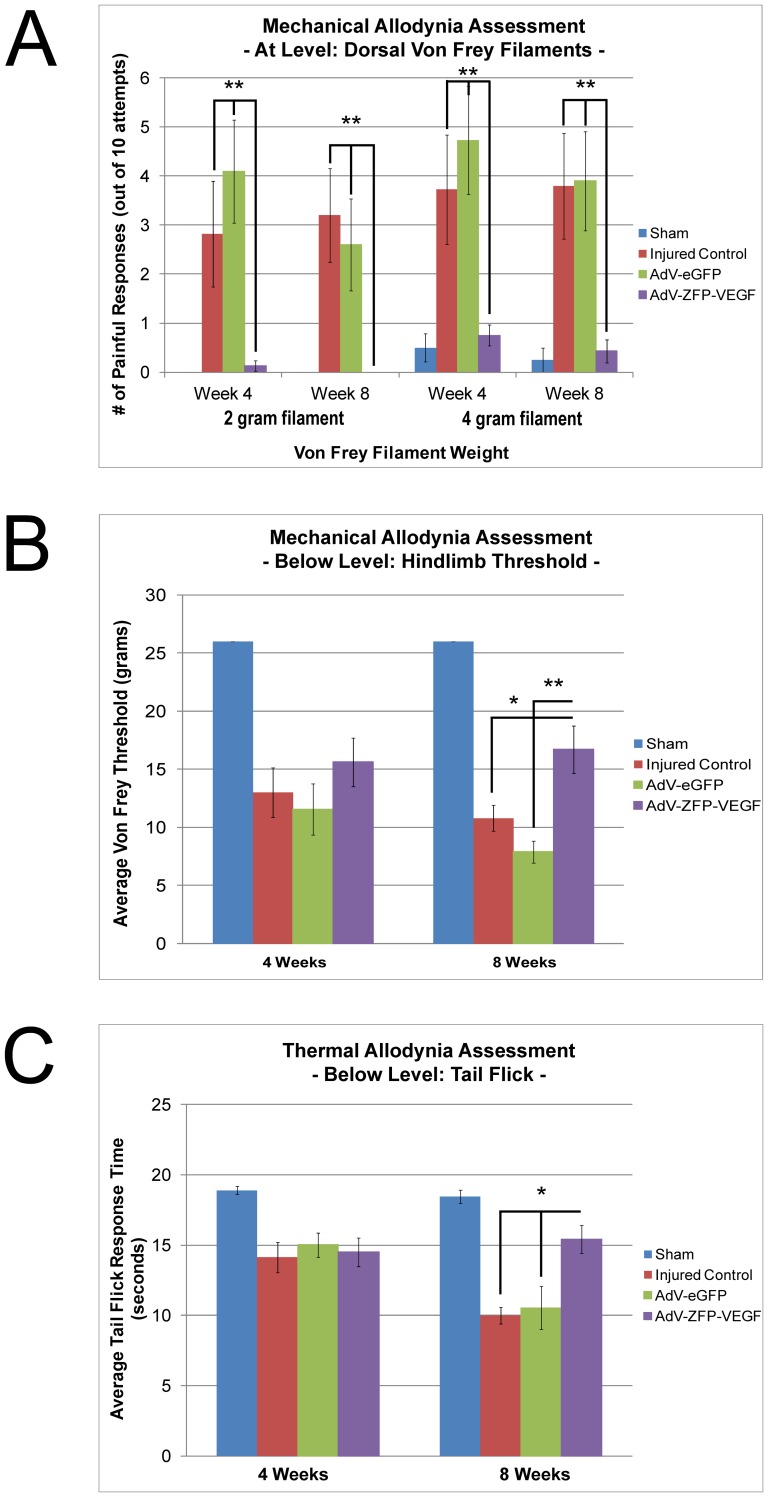
AdV-ZFP-VEGF significantly reduces mechanical and thermal allodynia at 8 weeks post-SCI. Mechanical and thermal allodynia, often used as outcome measures of neuropathic pain, were monitored with von Frey monofilaments and tail-flick tests, respectively. (A) At-level pain. Animals were assessed with 2 g or 4 g von Frey monofilaments around the dorsal incision (above T6–T7 laminectomy and injury). Data are expressed as the average number of adverse reactions out of 10 applications of the monofilament. There was an overall treatment effect with AdV-ZFP-VEGF using the 2 g and 4 g monofilaments at 4 weeks and 8 weeks post-injury; *p<0.05. (B) Below-level pain. Animals were subject to increasing von Frey filaments (2 g–26 g), and the when they elicited a response, this value was taken as the pain threshold value. Data is reported as the average threshold for each group. AdV-ZFP-VEGF increased hindlimb threshold compared to other injured groups; *p<0.05, **p<0.005. (C) Below-level thermal allodynia. A 50°C thermal stimulus was applied to the distal tip of the tail. The data shown is the average time it took for the animals to withdrawl their tail from the stimulus (“tail flick”). Shorter response times indicate a decreased pain threshold. Animals treated with AdV-ZFP-VEGF showed an increased tolerance/threshold to thermal stimuli at 8 weeks post-injury compared to other injured groups; *p<0.05. Data were analyzed by One-way ANOVA. Error bars represent SEM. n = 8/sham and AdV-ZFP-VEGF groups; n = 10/injured control and AdV-eGFP groups.

A devastating post-injury condition is neuropathic pain, which affects a significant portion of SCI patients [29], [30]. In this study we aimed to investigate the development of thermal and mechanical allodynia in AdV-ZFP-VEGF treated animals: hopeful that we would observe no increases in pain unlike the recent report by Nesic *et al.* [31]. Animals were tested for pain at 4 and 8 weeks following SCI, and here we observe that animals receiving AdV-ZFP-VEGF gene therapy have a significant reduction in allodynia, for both at-level and below-level pain, at 8 weeks post-injury (Figure 10). Testing with calibrated von Frey filaments around the lesion site (on the dorsal skin) showed AdV-ZFP-VEGF animals to have a significant reduction in at-level mechanical allodynia (Figure 10A; p<0.005). An increasing application of von Frey filaments to the plantar surface of the hindlimbs demonstrated a marked reduction in below-level alloydnia (Figure 10B), compared to injured control (p<0.05) and AdV-eGFP treated animals (p<0.005). Furthermore, we examined below-level thermal allodynia (Figure 10C), and observed a significant increase in pain tolerance (increased response time) in animals receiving AdV-ZFP-VEGF (p<0.05).

### AdV-ZFP-VEGF treatment results in spared grey matter, but not white matter tissue at 8 weeks post-SCI

**Figure 11:**
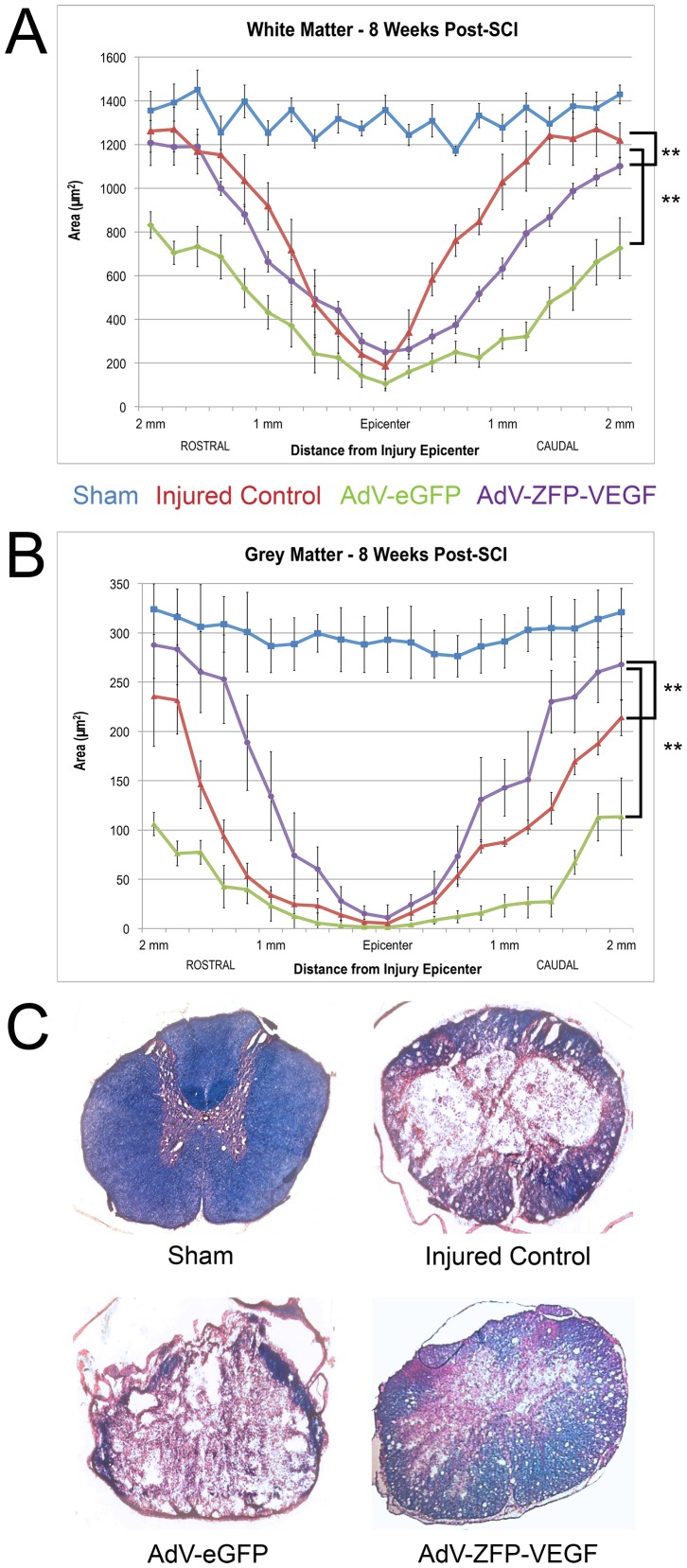
Tissue sparing quantification at 8 weeks post-SCI. (A) Residual white matter quantification. (B) Residual grey matter quantification. AdV-ZFP-VEGF improves spinal cord grey matter preservation. (C) Representative sections are shown from each group. Sections shown are taken 2 mm rostral to the epicenter at 8 weeks after SCI. AdV-ZFP-VEGF treated spinal cord exhibited a larger extent of grey matter spared tissue, but not white matter; **p<0.001. Data are mean ± SEM values. n = 8/sham and AdV-ZFP-VEGF groups; n = 10/injured control and AdV-eGFP groups.

Eight weeks after SCI, spinal cord cross-sections were stained serially with LFB-HE. Measurements of tissue sparing were calculated using StereoInvestigator software, and are expressed as the average cross-section area. Spinal cords from AdV-ZFP-VEGF treated rats did not show evidence of white matter tissue sparing compared to control injured animals (Figure 11A); however, AdV-ZFP-VEGF administration exhibited an overall increase in residual grey matter in (sections spanning 2 mm rostral and 2 mm caudal to the injury epicenter) when compared to tissue sections from AdV-GFP and injured control rats (Figure 11B; p<0.001). The grey matter differences observed in the AdV-ZFP-VEGF group are most notable in the peri-lesional area (1–2 mm rostral caudal to the epicenter). The epicenter of all injured and treated groups showed significant histological damage, and no differences were observed in tissue sparing at the lesion epicenter between the injured groups. Animals treated with AdV-eGFP show significantly reduced grey matter and white matter compared to injured control and AdV-ZFP-VEGF treated groups. As previously mentioned, we attribute the additional damage to the spinal cord in the AdV-eGFP group to the invasive delivery method of the treatment (intraspinal injections).

## Discussion

We have shown that AdV-ZFP-VEGF administration can be delayed 24 hours following spinal cord injury, and still provide beneficial effects. To date, we are the first to use AdV-ZFP-VEGF in a delayed fashion, and one of few studies that have used any form of VEGF therapy at a delayed point post-injury [32], [33]. In the current research, we chose to investigate the efficacy of 24 hour delayed AdV-ZFP-VEGF administration, which presents a clinically relevant therapeutic window. This form of gene therapy mimics physiological VEGF production, which should result in the production of all VEGF isoforms in the injured spinal cord: a necessary component for proper and functional angiogenesis. We observed significant improvements at the cellular and molecular levels, including an increased number of vessels, increased angiogenesis, reduced apoptosis and increased neurons. Additionally, we observed improved functional benefits in animals treated with AdV-ZFP-VEGF, including increased hindlimb weight support and significant reductions in allodynia. Collectively, the data suggest that: (i) administration of AdV-ZFP-VEGF results in an increase in VEGF, which may elicit effects through cell survival and angiogenic mechanisms (ii) AdV-ZFP-VEGF has a therapeutic time window following SCI which extends at least until 24 hours following injury, (iii) repairing vascular damage following neurotrauma is an important therapeutic target.

Previously, our lab has shown that immediate administration of AdV-ZFP-VEGF following SCI resulted in neuroprotection, increased vascular counts and improved functional recovery [11]. These promising results encouraged us to investigate a more feasible time-window for clinical intervention: 24 hours post-injury administration of AdV-ZFP-VEGF. Moreover, administration 24 hours following injury aimed to target a few important pathophysiological events post-SCI, particularly vascular damage and apoptosis. In a model of spinal cord contusion, Ling and Liu showed that TUNEL-positive cells are maximally observed at 48 hours following injury in both the grey and white matter [34]. Similarly, Crowe *et al.* demonstrated that maximal apoptosis is observed at 48 hours following contusion injury, with apoptosis identified between 6 hours and 3 weeks [35]. Liu *et al*. showed that following contusion injury TUNEL-positive neurons were observed between 4–24 hours, whereas TUNEL-positive glia were seen between 4 hours and 14 days with maximal numbers observed at 24 hours, although another peak of TUNEL-positive glia were observed at 7 days post-injury [36]. With respect to vascular targets, research suggests that angiogenic therapies should be administered to target endogenous vascular repair, which occurs between 3 and 7 days following injury [6], [37]. Therefore, by administrating AdV-ZFP-VEGF 24 hours following injury, we aimed to target and reduce apoptosis as well as enhance vascular regeneration. Our data, showing reduced TUNEL and increased endothelial cell proliferation, suggest that AdV-ZFP-VEGF is in fact capable of both neuroprotection and angiogenesis. Although we have not investigated the detailed mechanisms or signaling pathways of AdV-ZFP-VEGF *in vivo*, collectively our data provide strong evidence that increasing VEGF following injury may be beneficial and may stimulate cell survival and angiogenic pathways.

### Vasculature is a significant target following SCI

SCI results in significant vascular damage, including disruption of spinal cord blood flow, the onset of spinal cord ischemia, hemorrhage, edema and breakdown of the blood-spinal cord barrier (BSCB). These vascular changes encompass many of the earliest pathological processes following SCI, therefore therapies aimed directly at the vascular disruption or the ensuing downstream consequences of vascular injury are highly attractive; hence, we have chosen to address this therapeutic target in our research. In theory, rapid vascular repair following injury will likely result in the most favourable outcomes. Promoting repair and regeneration of vascular structures would mediate ischemia, hemorrhage and further edema by restoring proper blood-flow and stopping leaky vessels. Moreover, restoring the proper structure and function of the BSCB would likely reduce the influx of inflammatory cells into the spinal cord, thereby reducing the damage caused by reactive microglia [38], [39]. Recent reports have shown significant correlations between blood vessel density and improvements in recovery following CNS trauma [40]–[43]. Rescue and regeneration of the microvasculature within the epicenter and penumbra remains largely unexplored, yet may be a promising therapeutic route to facilitate tissue sparing and functional recovery following SCI. It has been shown that substantial trophic support is provided by CNS microvessels [44] and that microvessels are critical for tissue survival [45].

### VEGF is promising because it targets multiple cellular mechanisms

An immense number of cellular factors are involved in vascular development and repair, and in the pathological processes following SCI, therefore it is often suggested that the best therapy for SCI may involve a combinatorial approach. In the current research, VEGF was specifically selected since it has been shown to support the “neurovascular niche” and appears to play important roles in both vascular and nervous systems: bridging both endogenous systems. Expression of VEGF-R's have been observed in many cell types, including neurons, microglia/macrophages, endothelial cells, smooth muscle cells and astrocytes [46]–[51]. Through interactions with co-receptors, neuropilins, VEGF is able to influence the function and development of neural cells, which may be a key role for VEGF therapies following neurotrauma [52], [53]. Additionally, studies have shown that regenerating axons have a tendency to grow along blood vessels, therefore promoting vascular growth following injury may provide scaffolding for regenerating axons [54], [55].

### Previous studies using VEGF

In previous research that has used VEGF following SCI, authors have observed varying results. Choi *et al.* used a hypoxia-inducible VEGF-A expression system to treat rats with SCI and observed neuroprotective effects and enhanced VEGF-A expression [56]. Another group used an adenovirus coding for VEGF_165_, delivered via matrigel, in a partial spinal cord transection model. They observed a significant increase in vessel volume and a reduction in the retrograde degeneration of corticospinal tract axons [57]. However, Benton *et al*. [58] reported an exacerbation of lesion size and increased inflammation after the delivery of 2 µg of recombinant VEGF_165_ directly into the contused spinal cord 3 days post SCI. This study highlights several factors which are likely to be critical in the successful application of VEGF-A as a therapy for SCI. The method of VEGF delivery is likely a critical factor. In our study, we injected the ZFP-VEGF adjacent to the injury epicenter as the peri-lesional ischemic penumbra is likely the zone, which would benefit the most from approaches to enhance angiogenesis. Moreover, our delivery technique, using a ZFP-VEGF gene therapy, has the ability to upregulate several isoforms of VEGF-A (specifically we observed an upregulation of the VEGF 120, 164 and 188 isoforms), mimicking endogenous expression. In contrast, most other research has focused on the delivery of a single VEGF isoforms.

### Potential pitfalls of VEGF

Although VEGF has many desirable attributes for neuroprotection and vascular repair, it is important to recognize that some of these attributes have the potential to be deleterious and exacerbate damage following SCI. In development or maintenance of vascular structures, VEGF stimulates angiogenesis by signalling matrix-metalloproteinases (MMPs) to breakdown the BSCB and matrix in order to make way for new vascular sprouts. However, following injury, greater amounts of VEGF are released from surrounding cells and vascular remodelling is quickly initiated, which leads to a rapid hyperpermeability of the local vessels. This increase in permeability may contribute to an increased inflammatory response or increased edema following CNS injury. In particular, disruption of the BSCB following injury presents an entry route for inflammatory mediators to enter the CNS without resistance. A previous study reported that VEGF is able to promote monocyte migration *in vitro* and that administration of VEGF therapies may contribute to inflammatory responses following injury [59]. Although we observed no increased inflammatory response in our AdV-ZFP-VEGF animals compared to other injured control groups at 10 days following injury (data not shown), it is possible that VEGF therapies may exacerbate early inflammation.

### Disadvantages of intraspinal AdV injections

Although not perfect, direct injection into the spinal cord has some advantages and has been widely used for the delivery of therapeutics and stem cells [60]–[62]. Firstly, this method allows specific and localized delivery of the ZFP-VEGF gene therapy. We have selected four injections sites directly into the spinal cord, at 1 mm deep into the cord. This depth was selected to administer the vascular therapy close to the highly vascularized grey matter. Injections were administered two-millimeters rostral and caudal to the injury site to target the penumbra of the injury – a site which is more likely to be rescued by a delayed therapeutic intervention compared to the injury epicenter. Additionally, direct injections result in rapid delivery of the therapy, since it is not required to migrate or circulate before reaching the target tissue. In AdV-eGFP treated groups, we observed decreased NeuN counts, increased TUNEL-positive cells, a decrease in RECA-1-positive vessels, and diminished tissue sparing compared to animals which received only a compression injury (injured control group). We hypothesize that these deficits were likely attributed to additional damage caused by the intraspinal injections rather than exacerbated inflammation. Both AdV-eGFP and AdV-ZFP-VEGF groups received cyclosporine-A administration to minimize the inflammatory response, and we have previously shown that there is no difference in inflammation between AdV and control injured groups [11]. AdV-ZFP-VEGF treated animals were able to overcome these additional deficits and still show significant improvement compared to injured control animals, with the exception of white matter sparing. Direct injections (through the dorsal white matter) into the spinal cord may account for the data (Figure 11) that shows reduced white matter sparing for AdV-ZFP-VEGF treated animals compared to injured controls, since the injection likely caused additional physical insult to the spinal cord white matter. The administration of the therapy primarily aimed to rescue the vasculature following SCI, therefore the centrally located grey matter was the initial target and injections were given 1 mm deep into the cord. Although our data (Figure 1) indicate that AdV-ZFP-VEGF is observed in both the grey and white matter, AdV-ZFP-VEGF delivery was perhaps more localized to the deep grey matter tissue, and therefore able to exert the greatest effects on this population of cells (which would be supported by increased neuronal counts and vasculature; Figures 4 and 5, respectively).

In our studies, we believed it was important to include an AdV-eGFP control group to indicate the transduction of the virus *in vivo* (timing and location), and to help elucidate potential adverse effects of administering a gene therapy in an AdV construct. As noted above, the AdV-eGFP control group generally resulted in poorer outcomes compared to injured animals without injections. Future experiments may choose to include a saline-injected control group to more specifically determine the amount of damage caused by the injection verses the potential inflammatory response of an AdV vector. Moreover, additional studies should aim to investigate alternative delivery methods for AdV-ZFP-VEGF, as VEGF-treated animals may display even greater histological improvements over control animals if AdV-ZFP-VEGF were to be administered in a less invasive manner.

Future studies may also wish to examine the use of alternative viral or non-viral delivery methods. The use of AdV may induce a host inflammatory response, although we attempted to reduce these effects by the use of cyclosporine-A. However, AdV infection may result in an increase in inflammation, and increased inflammation may negatively contribute to the secondary injury. On the contrary, inflammation results in expression of cytokines and activation of matrix metalloproteinases, which ultimately drives angiogenesis and re-vascularization [63]. MMPs de-stablize vascular structures, allowing for vascular remodeling, and expression of pro-inflammatory cytokines actively recruits endothelial cells and promotes endothelial cell proliferation. AdV administration may play a role in inducing re-vascularization; however, our data do not indicate that AdV administration results in any substantial vascular benefits. In fact, we observed AdV-eGFP groups showing reduced vascular proliferation and overall vascular counts (Figure 5).

### Assessment of Functional Outcomes

We investigated the effects of 24 hour delayed AdV-ZFP-VEGF on the functional recovery and neuroanatomical preservation following thoracic SCI. In the current study, we used BBB locomotor scoring as well as CatWalk analysis to quantify functional outcomes of AdV-ZFP-VEGF therapy. CatWalk analysis is a relatively new method of assessing functional outcomes; however, the methodology has been widely validated for spinal cord injury models and generates data for many parameters of locomotion, which provides a more in-depth evaluation of functional recovery compared to traditional techniques [17]. CatWalk analysis demonstrated that animals treated with AdV-ZFP-VEGF showed improved locomotion as it was demonstrated by the increased forelimb stride length and the hindlimb swing speed. Interestingly, the animals treated with AdV-ZFP-VEGF exhibited improved hindlimb weight support. No differences were observed by BBB testing or electrophysiological assessment. Moreover, results indicated that AdV-ZFP-VEGF drastically reduced the development of mechanical and thermal allodynia in animals at 8 weeks post-injury. Lastly, results showed that AdV-ZFP-VEGF spared a significant amount of grey matter tissue compared to other injured groups.

The Basso Beattie Bresnahan (BBB) scoring scale to assess hindlimb deficits in thoracic SCI has been, and continues to be the “gold standard” for functional assessment [15]. The scoring system evaluates the hindlimb joint movement and the hind-paw orientation/stepping, provides a general indication of the locomotor capabilities of the animal, and establishes if the animal can weight-bear. The major shortcomings of the BBB are two-fold. First, although the BBB is to be conducted by blinded observers, the behavioural assessments are still highly subjective to human errors. Secondly – and perhaps the most confounding factor – the BBB is a qualitative system: simply indicating if the animal is competent of defined movements, providing a relatively subjective score of *how much* or *how well* an animal can perform a task (occasional, frequent or consistent). For detecting major functional differences in animals, the BBB scoring scale is highly effective and easy to conduct; however, more subtle differences between treatment groups may not be observed by BBB assessment. Additionally, since the BBB scale is not a linear relationship between the numerical value and the functional gains associated with them, teasing out meaningful results can become a challenge.

In this research, we used both the BBB and the Catwalk gait analysis software to assess functional recovery post-injury. While scoring animals using BBB, subtle differences between animals were noted (some of them moved more normally, and with greater consistency); however, these variations were not strong enough to increase their BBB score. Overall, we observed no differences in BBB scores between groups. On the other hand, Catwalk data indicated that AdV-ZFP-VEGF treated animals have significant improvements in hindlimb weight support, and hindlimb swing speed. It should be emphasized that CatWalk experiments require weight support, and therefore only a sub-set of animals (n = 5) for each group were tested using the Catwalk system. The average BBB scores for the injured control and AdV-ZFP-VEGF animals used for CatWalk experiments were very similar (injured control  = 9.5, AdV-ZFP-VEGF = 9.2), whereas the AdV-eGFP animals had poorer BBB scores and the majority of animals did not reach weight-bearing ability (average BBB score  = 7.9). Although the BBB is a valid, widely used method of behavioural evaluation, the Catwalk is a more sensitive and quantitative outcome measure, which may reveal understated changes in recovery not observable using the BBB scoring system. From a clinical perspective, improvements in hindlimb weight support and in overall locomotion may have an important impact on the mobility and independence of an injured individual. Interestingly, Catwalk analysis also revealed that AdV-ZFP-VEGF animals showed improved forelimb stride length, suggesting that AdV-ZFP-VEGF could potentially enhance hindlimb-forelimb coordination; although with BBB scores of 9, we did not observe hindlimb-forelimb coordination in any injured animal group. In the histological examination of grey and white matter post-SCI, we did not investigate sparing of specific pathways or specific neuronal phenotypes (i.e. interneurons vs. motor neurons); however, improvements in both hindlimb and forelimb kinetics could suggest that AdV-ZFP-VEGF may spare propriospinal interneurons, which are located at the grey-white matter interface and are involved in coordination of limb movements [64]. Future experiments involving AdV-ZFP-VEGF should aim to investigate the effects of AdV-ZFP-VEGF on interneuron sparing/survival, since these cells have been attributed to regulating central pattern generators (CPGs) and should therefore be of interest for promoting locomotor recovery following SCI.

In compliment to our data showing AdV-ZFP-VEGF spares neurons, in this study we quantified residual tissue at 8 weeks post-SCI and our data show that delayed AdV-ZFP-VEGF administration results in improved grey matter sparing, but not white matter sparing. Taken together, these results suggest that AdV-ZFP-VEGF likely acts by promoting survival of neuronal cell bodies. Additionally, we observe that AdV-eGFP animals show notable decreases in both grey and white matter sparing at 8 weeks post-SCI. We do observe the caudal sections to have even less tissue sparing; however, it is often observed that tissue caudal to a traumatic injury is less preserved due to Wallerian degeneration, axonal disruption and reduced vascular flow. In a previous study, we show that AdV administration (in conjunction with cyclosporine-A) does not result in an increased inflammatory response compared to injured control animals [11]. The functional outcomes observed in our study – improved gait, hindlimb weight support and decreased pain – would be consistent with previous research demonstrating improved function and/or sparing of propriospinal tracts (limb coordination) [65], reticulospinal tracts (locomotion and weight-bearing stepping) [66], and spinothalamic tracts (neuropathic pain) [67], [68] following SCI. In our study, we did not investigated the sparing of specific spinal tracts via electrophyisiology or dye-tracing experiments; however, an overall increase in residual grey matter likely contributes to improved pain processing pathways (interneurons) and a decrease in aberrant pain [17], [69], [70]. Varying studies report that 26-96% of human patients experience neuropathic pain following SCI [30]. Research has identified VEGF as one of the potential factors involved in the development of neuropathic pain; however, it is still unclear if VEGF plays a beneficial or detrimental role. Schratzberger *et al*., found that intramuscular injections of VEGF improved vascularity, blood flow and peripheral nerve function in a rabbit model of diabetic neuropathy [71]. Since it is believed that diabetic neuropathy is caused from microvascular ischemia, their findings reasonably support the use of VEGF for the treatment of neuropathies. Conversely, Nesic *et al*. recently showed that VEGF administration into the spinal cord resulted in an increased number of animals displaying neuropathic pain, as well as an increase in myelinated dorsal horn neurons, suggesting that VEGF results in non-specific axonal sprouting [31]. Regardless of whether VEGF therapies result in favourable or damaging outcomes, it is most important for future research to be aware of potential pitfalls of VEGF administration and to consider the implications they may have on the bench-to-bedside translation of these therapies. Future studies are required to investigate the exact mechanisms of the attenuated allodynia/neuropathic pain observed following AdV-ZFP-VEGF administration.

## Conclusions

The present data demonstrate that, similar to the effects seen following immediate administration of AdV-ZFP-VEGF shown by Liu *et al.*, treated animals show increased VEGF mRNA and protein levels, increased vascular counts, increased neuroprotection and reduced apoptosis [11]. Overall, the administration of AdV-ZFP-VEGF shows promise as a therapeutic treatment for SCI, and these findings suggest that AdV-ZFP-VEGF treatment can be delayed up to 24 hours following injury, which presents a feasible time-window for clinical intervention. To the best of our knowledge, we are the first to investigate the delayed administration of AdV-ZFP-VEGF in a model of SCI. Here we observe beneficial effects in a variety of cell populations, and show that these cellular outcomes appear to be translated into improved functional recovery, as well as attenuated allodynia following SCI. Overall, these data suggest that targeting vascular and neuroprotective mechanisms by AdV-ZFP-VEGF administration may be a viable treatment for spinal cord injury. Collectively, this research further supports the use of VEGF as a potential candidate for neurotrauma treatments.
